# Secondary aneurysmal bone cyst in the distal humerus after resection of intra-articular nodular fasciitis of the elbow

**DOI:** 10.1186/s13104-015-1279-5

**Published:** 2015-07-22

**Authors:** Michiro Yamamoto, Hiroshi Urakawa, Yoshihiro Nishida, Hitoshi Hirata

**Affiliations:** Department of Hand Surgery, Nagoya University Graduate School of Medicine, 65 Tsurumai-cho, Showa-ku, Nagoya, 466-8550 Japan; Department of Orthopaedic Surgery, Nagoya University Graduate School of Medicine, 65 Tsurumai-cho, Showa-ku, Nagoya, 466-8550 Japan

**Keywords:** Aneurysmal bone cyst, Intra-articular nodular fasciitis, Elbow

## Abstract

**Background:**

Nodular fasciitis most often occurs within subcutaneous tissues, but may also arise within skeletal muscle, dermis, vessels, peripheral nerves and, although rarely, within joints. Knowledge regarding the cause of aneurysmal bone cysts, its natural history, and the results of treatment is limited. Secondary aneurysmal bone cysts are associated with other neoplastic processes. Intra-articular nodular fasciitis in the elbow joint has not been reported previously, nor has the development of aneurysmal bone cyst secondary to intra-articular nodular fasciitis in the elbow joint.

**Case presentation:**

We report an unusual case of a Japanese 13-year-old boy who presented with a 1-year history of right elbow pain. The onset of pain was insidious, without antecedent trauma. On physical examination, the range of motion of the elbow was limited. Grip strength was reduced in the affected extremity. Incisional biopsy was performed and histologic findings revealed nodular fasciitis in the elbow joint. After tumor excision, a secondary aneurysmal bone cyst in the distal humerus developed. Endoscopy-assisted curettage and artificial bone grafting were performed. One year after surgery, a plain radiography showed no recurrence, and the patient returned to his daily activities without any symptoms.

**Conclusion:**

An aneurysmal bone cyst in the distal humerus developed after excision of intra-articular nodular fasciitis arising in the elbow. The secondary aneurysmal bone cyst successfully healed after endoscopy-assisted curettage and artificial bone grafting. The findings of this case suggest that these two tumors reside in the same biologic spectrum defined as *USP6*-induced tumors.

## Background

Nodular fasciitis is a benign, self-limiting proliferation of fibroblasts with a preference for the upper extremities, trunk, and head and neck of young adults. Konwaler et al. first reported this disease as subcutaneous pseudosarcomatous fibromatosis in 1955 [1]. Nodular fasciitis most often occurs within subcutaneous tissues, but may also arise within skeletal muscle, dermis, vessels, and peripheral nerves, and rarely within joints [2–5].

Aneurysmal bone cysts are also benign, locally aggressive bony lesions that were first commented on by Jaffe and Lichtenstein in 1942 [6]. Primary aneurysmal bone cysts comprise approximately 70% of all aneurysmal bone cyst diagnoses. Secondary aneurysmal bone cysts can develop in association with other neoplastic processes [7]. An aneurysmal bone cyst is the result of a specific pathophysiologic change which is probably the consequence of either trauma or a tumor-induced anomalous vascular process [8].

Recently, Oliveira and Chou suggested that aneurismal bone cysts and nodular fasciitis reside in the same biologic spectrum as *USP6*-induced tumors [9].

Herein, we present a case of secondary aneurysmal bone cyst in the distal humerus after resection of intra-articular nodular fasciitis within the elbow joint.

## Case presentation

A Japanese 13-year-old boy presented with a 1-year history of right elbow pain. The onset of pain was insidious, without antecedent trauma. He reported pain on both flexion and extension of the elbow. He had no medical history of disease, and his family had no history of musculoskeletal disease. On physical examination, flexion and extension of the elbow was limited to a range of 116°–30°. Grip strength was reduced in the affected extremity to 16 kg, compared to 21 kg on the left. Neurovascular examination showed normal results, with no lymphadenopathy or other enlargements.

**Figure 1:**
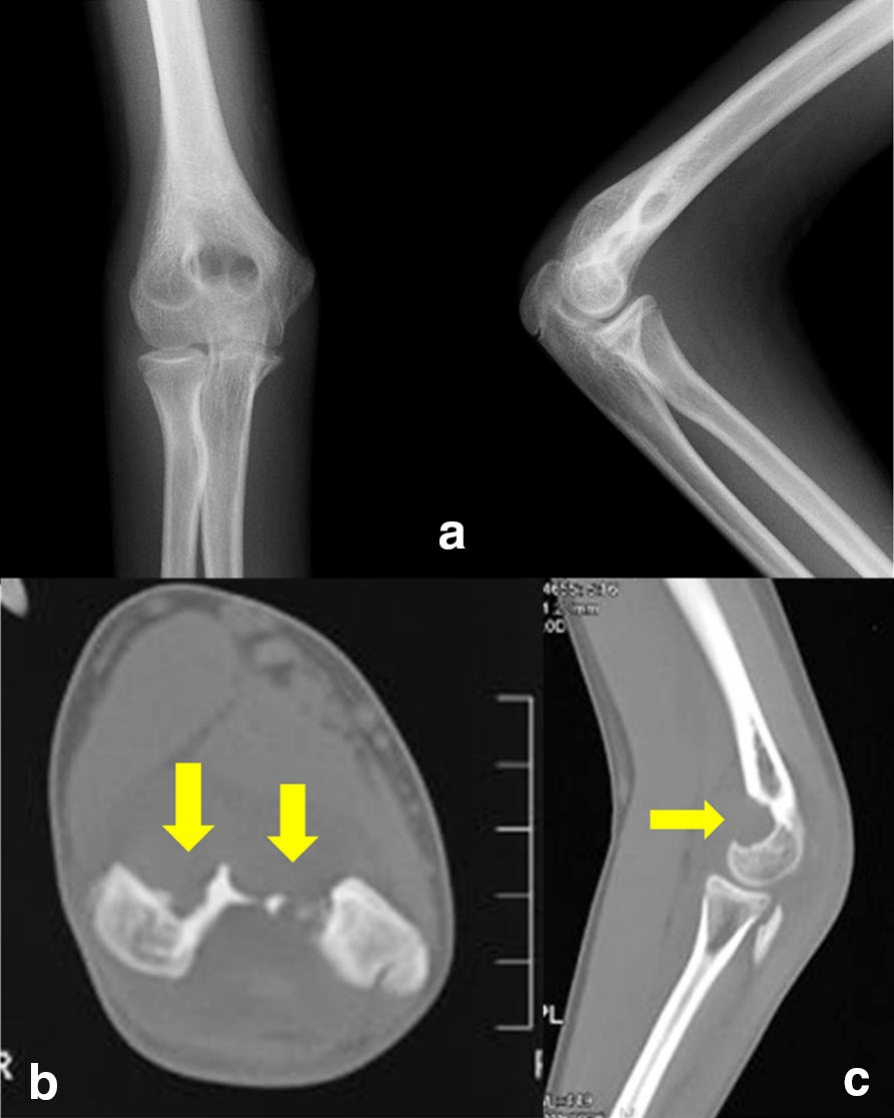
Plain radiography and computed tomography (CT) findings on admission. Plain radiography (**a**), axial CT (**b**) and sagittal CT (**c**) of the *right* elbow shows scalloping (*yellow arrow*) of the anterior portion of the distal humerus.

**Figure 2:**
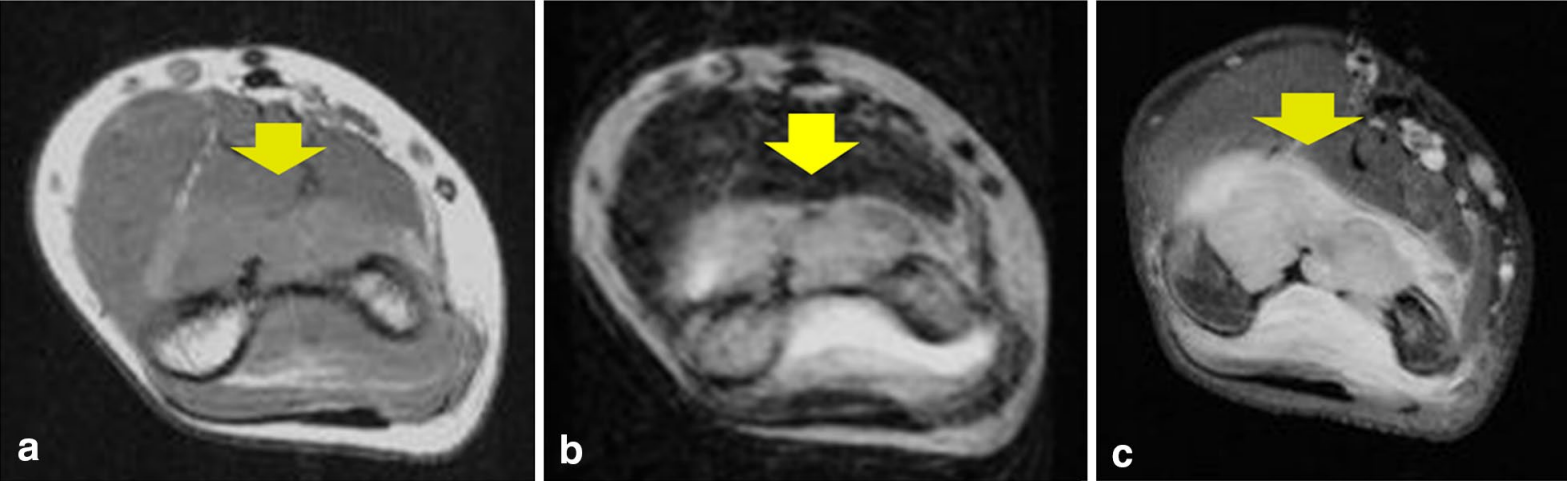
Magnetic resonance imaging (MRI) findings on admission. MRI shows multiple soft masses measuring a total of 3 × 2 × 2 cm in the anterior portion within the elbow joint (*yellow arrow*). The lesion is isointense with muscle on T1-weighted MRI (**a**), and predominantly hyperintense on T2-weighted imaging with fluid effusion in the posterior elbow joint (**b**). Post-gadolinium sequences show no foci of blooming that would suggest hemosiderin deposition. Thick, peripheral and septal enhancement is seen of not only the masses, but also the whole elbow joint (**c**).

Plain radiography and computed tomography (CT) of the right elbow showed scalloping of the anterior portion of the distal humerus (Figure 1a–c). Magnetic resonance imaging (MRI) showed multiple soft masses measuring 3 × 2 × 2 cm in the anterior portion within the elbow joint. The lesion was isointense with muscle on T1-weighted MRI (Figure 2a), and predominantly hyperintense on T2-weighted imaging with fluid effusion in the posterior elbow joint (Figure 2b). Post-gadolinium sequences showed thick, peripheral, and septal enhancement that was evident for not only the masses, but also the whole elbow joint (Figure 2c). Pigmented villonodular synovitis, localized nodular synovitis, and complex ganglion were suggested as possible differential diagnoses. However, the possibility of soft tissue sarcoma could not be excluded. Incisional biopsy was performed under local anesthesia and histologic findings revealed nodular fasciitis in the elbow joint.

**Figure 3:**
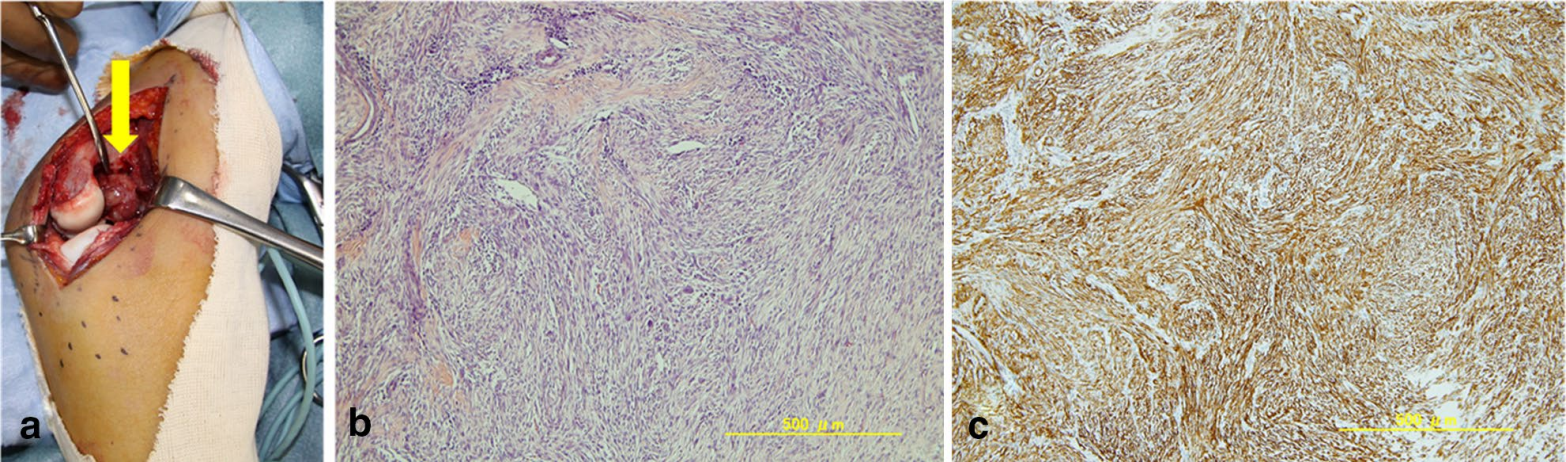
Surgical, pathological, and immunohistochemistry findings. After incision of the lateral capsule of the elbow, the encapsulated mass (*yellow arrow*) was exposed and excised (**a**). Pathological examination revealed typical histological features of nodular fasciitis, comprising cytologically bland, uniform plump spindle cells arranged in short intersecting bundles within a collagenous stroma (**b**). Immunohistochemistry shows diffuse positivity for smooth muscle actin (**c**).

We performed excisional biopsy under general anesthesia. After incision of the lateral capsule of the elbow, the encapsulated mass was exposed and excised (Figure 3a). Part of the tumor existed within the distal humerus, so curettage was also performed. The lateral collateral ligament complex was repaired with nylon 4-0 suture. After surgery, a compression bandage was applied to the elbow for 2 weeks, and then active and passive range of motion exercises commenced under the supervision of hand therapists. Pathological examination proved typical histological features of nodular fasciitis, comprising cytologically bland, uniform plump spindle cells arranged in short, intersecting bundles within a collagenous stroma (Figure 3b). Immunohistochemistry showed that the sample was diffusely positive for smooth muscle actin (SMA) (Figure 3c), but negative for S-100, desmin, CD34, anti-cytokeratin (CAM5.2), pan-cytokeratin antibody (AE1/AE3), and anaplastic lymphoma kinase. Symptoms of the elbow gradually resolved and the patient returned to normal activities after excisional biopsy.

**Figure 4:**
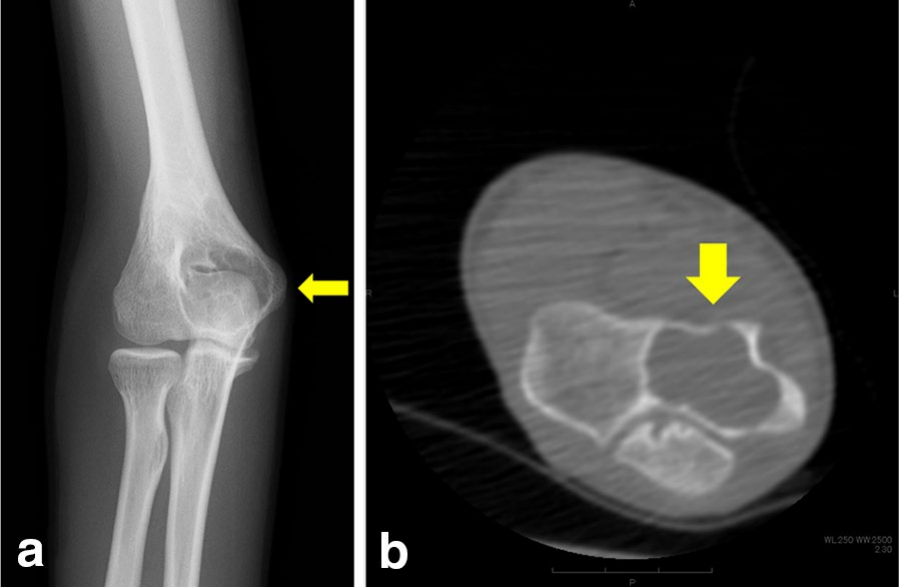
Follow-up plain radiography and computed tomography (CT) findings. At 1 year after excisional biopsy, plain radiography (**a**) and CT (**b**) show a large bone cyst in the distal humeral epiphysis (*yellow arrow*).

**Figure 5:**
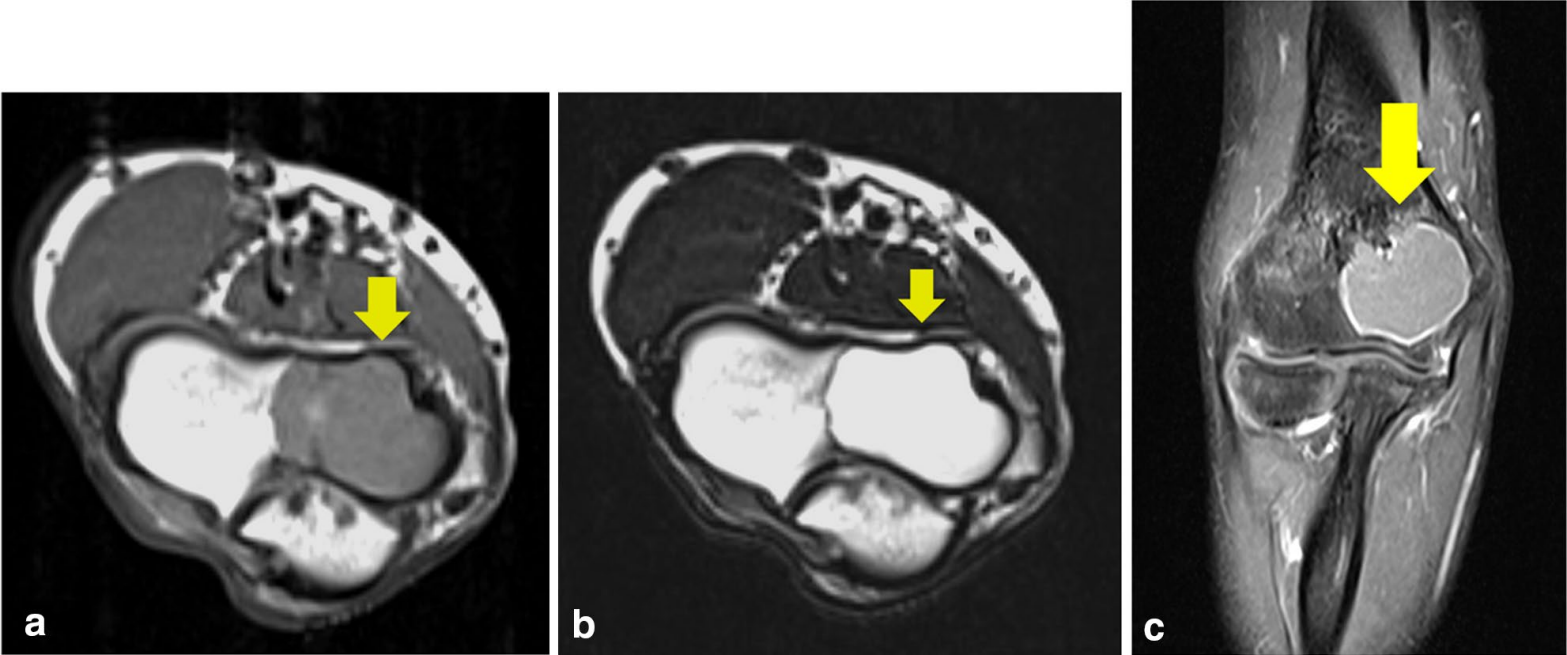
Follow-up magnetic resonance imaging (MRI) findings. The lesion appears hypointense to muscle on T1-weighted MRI (**a**), and predominantly hyperintense on T2-weighted fat-saturated sequences (**b**). Post-gadolinium sequences show peripheral enhancement of the cyst (**c**). MRI reveals simple bone cyst or aneurysmal bone cyst in the distal humerus (*yellow arrow*).

**Figure 6:**
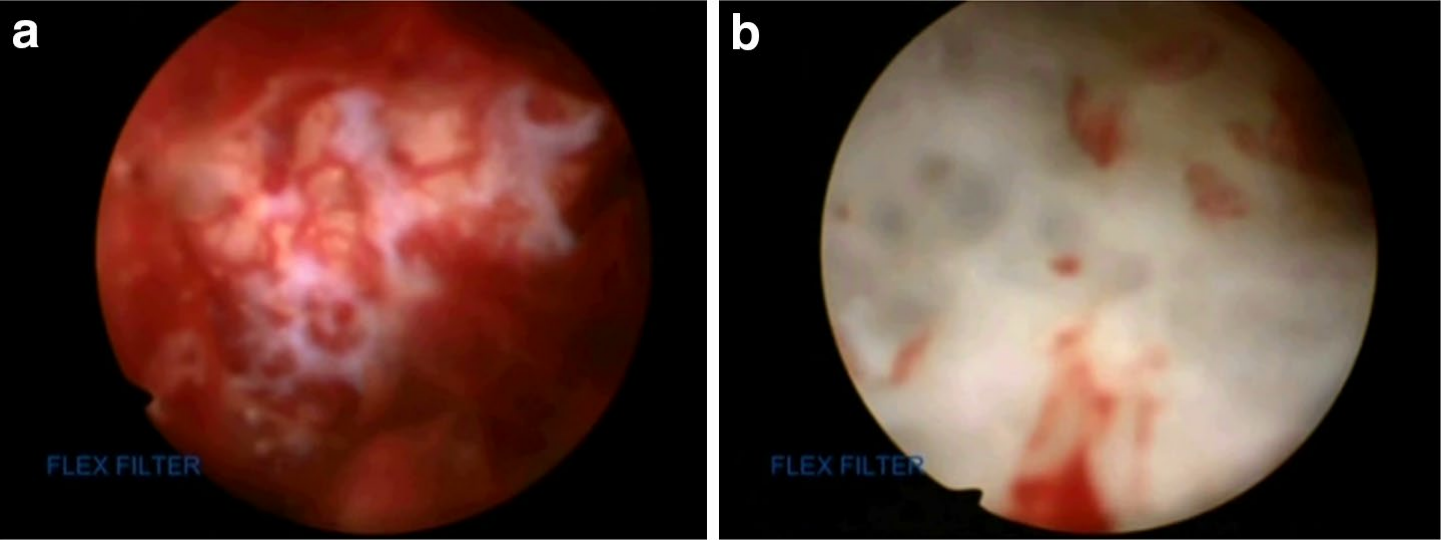
Views from the endoscopy of the bone cyst. The structure within the cyst is shown before curettage (**a**) and after curettage (**b**).

**Figure 7:**
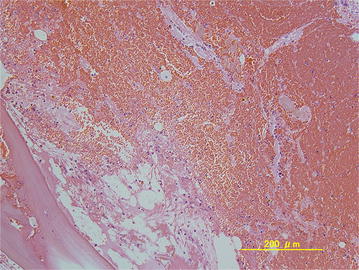
Pathological examination of the curettage sample. Pathological examination of the specimen obtained by curettage shows aneurysmal bone cyst. The blood-filled chamber is irregular in structure, with islands of bone and fibrous tissues.

**Figure 8:**
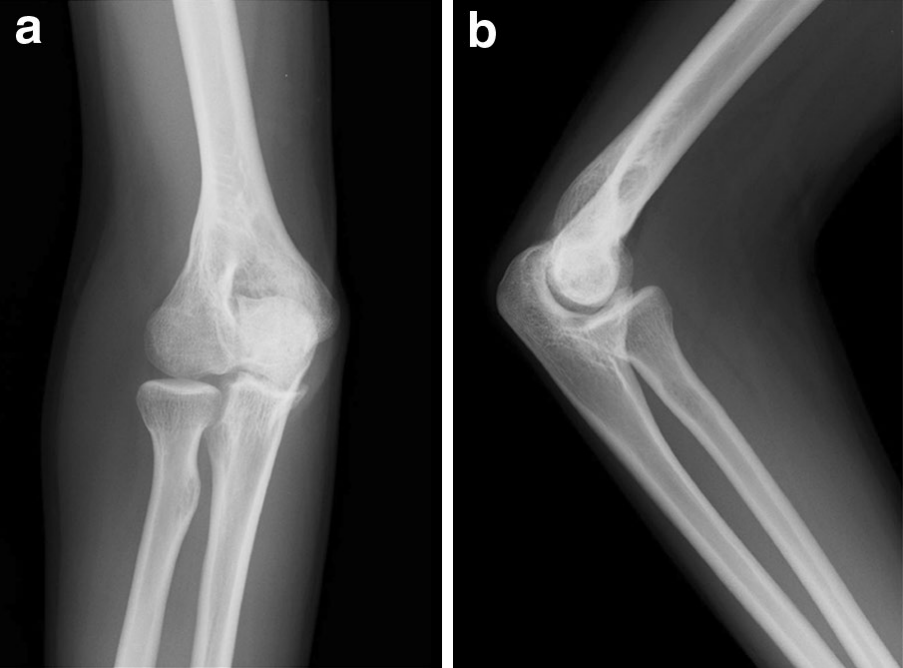
Plain radiography findings 1 year after curettage and artificial bone grafting. One year after curettage and artificial bone grafting, plain radiography of antero-posterior (**a**) and lateral (**b**) views show no recurrence. The patient returned to daily activities without any symptoms.

At 1 year postoperatively, plain radiography and CT revealed a large bone cyst in the distal humeral epiphysis (Figure 4a, b). The lesion was hypointense to muscle on T1-weighted imaging (Figure 5a), and predominantly hyperintense on T2-weighted fat-saturated sequences (Figure 5b). Post-gadolinium sequences showed peripheral enhancement of the cyst (Figure 5c). MRI revealed either a simple bone cyst or an aneurysmal bone cyst in the distal humeral epiphysis. Endoscopy-assisted curettage and artificial bone grafting (OSferion; Olympus Terumo Biomaterials, Tokyo, Japan) were performed under general anesthesia. Figure 6a, b shows the views from the endoscopy of the bone cyst. Pathological examination of the curettage sample showed an aneurysmal bone cyst. The blood-filled chamber was irregular in structure, with islands of bone and fibrous tissues. Immature bone formations were identified along with the fibrous septa (Figure 7). One year after curettage and artificial bone grafting, plain radiography showed no recurrence (Figure 8a, b). On physical examination, flexion and extension of the elbow was recovered to a range of 134°–0°. Grip strength was also recovered in the affected extremity to 31 kg, compared to 30 kg on the healthy side. The patient returned to his daily activities without any symptoms.

## Discussion

Intra-articular nodular fasciitis is a rare pathology. Only one case of nodular fasciitis of the elbow has been described previously, but no tumor was present within the elbow joint [10]. Hornick et al. reported 10 cases of intra-articular nodular fasciitis, with seven lesions arising in the knee, two in the hand, and one in the ankle. On immunohistochemical examination, all cases appeared diffusely positive for SMA. The histological features are basically the same as nodular fasciitis, except for the relatively frequent presence of extensive stromal hyalinization and other degenerative changes [5].

Knowledge regarding the cause of aneurysmal bone cyst, its natural history, and the results of treatment is limited. In prior studies, findings have suggested the possibility that aneurysmal cyst is actually a result of hemorrhagic degradative events occurring in patients with other lesions including giant cell tumor, hemangioma, chondroblastoma, osteoblastoma, non-ossifying fibroma, fibrous dysplasia, chondromyxoid fibroma, eosinophilic granuloma, and other tumors [6, 7]. The association of not only neoplasm, but also trauma was noted by both Jaffe and Lichtenstein [11, 12]. According to Mirra et al., the so-called aneurysmal bone cyst is neither a cyst nor a neoplasm; rather, it is probably a periosteal to intraosseous arteriovenous malformation, not uncommonly seen in association with other well-known benign and even malignant lesions [13]. Recently, the biological spectrum of aneurysmal bone cyst and nodular fasciitis was reported. Aneurismal bone cyst and nodular fasciitis are characterized by structurally similar *USP6* fusion genes. USP6 is a ubiquitin-specific protease that was identified as an oncogene in transfection experiments with Ewing sarcoma DNA two decades ago. The identification of structurally similar *USP6* fusion genes in both the aneurismal bone cyst and nodular fasciitis suggested that these are clonal neoplastic disorders that may belong to the same biologic spectrum [9]. In the present case, two mechanisms for the development of secondary aneurismal bone cyst formation are considered. One is that the periosteal to intraosseous arteriovenous malformation might have developed after excision of the intra-articular nodular fasciitis and curettage of the distal humeral lesion. Then, a secondary aneurysmal bone cyst of the distal humeral epiphysis may have developed in the remodeling process after surgery. The other pathomechanism involves the similar *USP6* fusion genes. The site of nodular fasciitis was mainly in the radial side. However, the aneurismal bone cyst was present only in the ulnar side of the distal humerus. We cannot explain the pathogenesis only by the remodeling process after surgical intervention. Although we did not perform the molecular diagnosis, this case strongly suggests that these two tumors reside in the same biologic spectrum defined as *USP6*-induced tumors.

Mankin et al. reviewed 150 patients with aneurysmal bone cyst. The recurrence rate after various treatments including curettage and insertion of bone graft or polymethylmethacrylate was 20% [7]. In another series of 96 cases, the overall rate of recurrence of aneurysmal bone cyst after surgery including curettage and adjuvant treatment was 11.5%. The rate of recurrence was 20.6% after curettage and high-speed-burr treatment alone and 7.5% after curettage and high-speed-burr treatment added to argon beam coagulation [14]. In this case, endoscopy-assisted curettage was successfully performed. One year after curettage and artificial bone grafting, plain radiography showed no recurrence.

## Conclusion

We have reported an unusual case of intra-articular nodular fasciitis arising in the elbow and developing to aneurysmal bone cyst after tumor excision. Endoscopy-assisted curettage was successfully performed. The site of nodular fasciitis was mainly in the radial side, while the aneurismal bone cyst was present only in the ulnar side of the distal humerus. The pathogenesis cannot be explained only by the remodeling process after surgery. Although the molecular diagnosis was not performed, the findings of present case suggest that these two tumors reside in the same biologic spectrum defined as *USP6*-induced tumors.

## Consent

Written informed consent was obtained from the patient and his father for publication of this Case Report and any accompanying images. A copy of the written consent is available for review by the Editor-in-Chief of this journal.

## Abbreviations

CT: computed tomography
MRI: magnetic resonance imaging
SMA: smooth muscle actin
